# ATP as a Pathophysiologic Mediator of Bacteria-Host Crosstalk in the Gastrointestinal Tract

**DOI:** 10.3390/ijms19082371

**Published:** 2018-08-12

**Authors:** Akie Inami, Hiroshi Kiyono, Yosuke Kurashima

**Affiliations:** 1 Department of Mucosal Immunology, IMSUT Distinguished Professor Unit, The Institute of Medical Science, The University of Tokyo, Tokyo 108-8639, Japan; usajiro38@gmail.com (A.I.); kiyono@ims.u-tokyo.ac.jp (H.K.); 2 International Research and Development Center for Mucosal Vaccines, The Institute of Medical Science, The University of Tokyo, Tokyo 108-8639, Japan; 3 Division of Gastroenterology, Department of Medicine, CU-UCSD Center for Mucosal Immunology, Allergy and Vaccines, University of California, San Diego, CA 92093-0956, USA; 4 Department of Immunology, Graduate School of Medicine, Chiba University, Chiba 260-8670, Japan; 5 Mucosal Immunology and Allergy Therapeutics, Institute for Global Prominent Research, Graduate School of Medicine, Chiba University, Chiba 260-8670, Japan; 6 Department of Mucosal Immunology, Graduate School of Medicine, Chiba University, Chiba 260-8670, Japan; 7 Department of Innovative Medicine, Graduate School of Medicine, Chiba University, Chiba 260-8670, Japan

**Keywords:** ATP, adenosine, inflammatory bowel disease (IBD), purinergic pathway, inter-species communication, commensal bacteria

## Abstract

Extracellular nucleotides, such as adenosine triphosphate (ATP), are released from host cells including nerve termini, immune cells, injured or dead cells, and the commensal bacteria that reside in the gut lumen. Extracellular ATP interacts with the host through purinergic receptors, and promotes intercellular and bacteria-host communication to maintain the tissue homeostasis. However, the release of massive concentrations of ATP into extracellular compartments initiates acute and chronic inflammatory responses through the activation of immunocompetent cells (e.g., T cells, macrophages, and mast cells). In this review, we focus on the functions of ATP as a pathophysiologic mediator that is required for the induction and resolution of inflammation and inter-species communication.

## 1. ATP as an Inter-Species Messenger

Although adenosine-5′-triphosphate (ATP) is generated intracellularly through glycolysis and the tricarboxylic acid cycle, it also functions in the extracellular compartment [1,2]. The action of extracellular ATP (eATP) in the mammalian intestine was first described in 1934 [3], and it is now known that eATP released from nerve termini, immune cells, injured or damaged cells, and some commensal bacteria plays important roles in inter-species communications [4,5,6]. Several eATP release pathways, including the constitutive secretory pathway, the activation of P2Y receptor (one of purinergic receptors)-mediated Ca^2+^-regulated exocytosis, P2X7 receptor (P2X7R) channels, and conductive release through pannexin 1 (Panx1) and connexin (Conx) membrane hemichannels, were previously reviewed [1,2,7,8,9]. In the gut, eATP is released from activated immune cells and injured or damaged epithelial cells [10,11,12]. For example, chemotactic mediators, such as interleukin 8 (IL-8), leukotriene B4, and the complement product C5a, prompt the release of eATP from neutrophils, and thus, enhance their migratory ability in a paracrine and autocrine manner [13,14]. Similar autocrine signaling systems were identified in macrophages [15]. In humans, the stimulation of formyl-peptide, Fcγ, IL-8, complement C5a, and leukotriene B4 receptors on neutrophils leads to the release eATP through Panx1 channels [14]. In T-cells, activation of the T-cell receptor and the cluster of differentiation 28 (CD28) co-stimulatory receptor promotes Panx1-induced eATP secretion [16,17]. In addition to these hemichannel-dependent eATP release pathways in various cells types, a unique eATP-amplifying system is present in mast cells. Once mast cells are stimulated by allergens or eATP, they release eATP, which is amplified through the activities of ecto-adenylate kinase and ATP synthase even though ATP is concurrently metabolized to ADP, AMP, and adenosine by ecto-nucleotidases [18].

**Figure 1:**
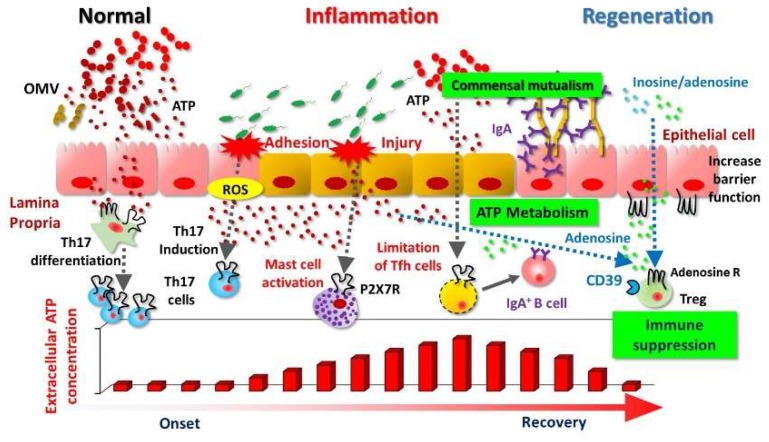
ATP as a pathophysiologic mediator for bacteria-host communication. The pathways through which extracellular ATP (eATP) mediates communication between bacteria and host immune cells in the intestinal compartment are shown. Bacteria (e.g., *Escherichia coli*) produce ATP in a growth-phase-dependent manner and secrete it in outer membrane vesicles (OMVs). ATP derived from commensal bacteria leads to the differentiation of intestinal T helper 17 (Th17) cells through the stimulation of antigen-presenting cells. The reactive oxygen species (ROS) produced by adhesion of bacteria to epithelial cells promotes differentiation of Th17 cells. ATP activates mast cells and enhances inflammatory responses via P2X7 (e.g., chemical mediator release and inflammatory cell infiltration). In the small intestine, eATP released by commensal bacteria indirectly limits immunoglobulin A (IgA) responses to various bacteria by interacting with P2X7 on follicular helper T cells (Tfh cells), thus decreasing Tfh cell numbers. Purine metabolites, adenosine or inosine, inhibit inflammatory responses through interaction with adenosine receptors (e.g., A_2A_R.). The dotted line arrow shows the eATP acts immune cells. The regular arrow shows induction of IgA producing B cells via cytokine production from Tfh cells.

In addition, commensal bacteria are another major source of eATP. The levels of eATP increase dramatically as bacteria exit the stationary phase [19], peak around the end of the log-phase of growth, and decrease again as they approach stationary phase [20]. In the small intestine, ATP derived from commensal bacteria restricts the immunoglobulin A (IgA) response directed against them by limiting the number of follicular helper T (Tfh) cells, which regulates the maturation and activation of mucosal IgA antibody production; disruption of ATP signaling increases the number of Tfh cells in the gut [6]. This finding indicates that bacterial eATP influences the host mucosal immune system and subsequently regulates the bacterial community, thus promoting a proficient gut ecosystem [6] (Figure 1). In this regard, Iwase and colleagues reported that *Enterococcus gallinarum*, a vancomycin-resistant Gram-positive coccus isolated from mice and humans, secretes ATP [21]. These authors subsequently identified seven additional eATP-secreting enterococcal species and determined that glycolysis is the most important pathway for bacterial ATP secretion [22]. Their studies indicate that diverse bacteria have the potential to secrete eATP, especially during the growth phase and in the presence of glucose [22]. In addition, kinetics investigations revealed that exposure to amphipathic peptides (e.g., melittin, and mastoparan 7), which enhance membrane permeability to small solutes in a variety of cells, also increases the release of eATP from *Escherichia coli* [23]. In *E. coli*, eATP is packed into nano-scaled membrane particles, called outer membrane vesicles (OMVs) [23]. These OMVs are continuously formed by *E. coli* under normal growth conditions (Figure 1). Furthermore, eATP acts as a cross-communication agent between bacteria and their host and as an energy-exchange partner to facilitate the formation and survival of bacterial communities [20].

In the host immune system, eATP contributes to the initiation and activation of immune responses. Specifically, eATP mediates the “find-me” signal from injured or dead cells, and acts as a danger signal that elicits a variety of inflammatory responses [24,25]. For example, T helper 17 (Th17) cells are induced by eATP signaling, which is mediated by antigen-presenting cells, and are presumed to be important factors in the pathogenesis of inflammatory bowel disease (IBD) [5,26,27] (Figure 1). Furthermore, eATP derived from commensal bacteria drives the differentiation of intestinal Th17 cells, and the administration of ATP to mice exacerbates T-cell-mediated enterocolitis [5,28].

The reactivity of relevant cells and their responses (such as proliferation, migration, and activation; cell death; maintenance of homeostasis; and initiation of inflammation) differ depending on the eATP concentration and reactive receptors [29,30]. The eATP receptors, known as purinergic receptors, are divided into two types: P1, which binds adenosine, and P2, which binds nucleotides such as ADP/ATP and uridine diphosphate/triphosphate (UDP/UTP) [31,32,33,34]. The P1 adenosine receptors are G-protein-coupled (metabotropic) receptors comprising four subtypes (A_1_, A_2A_, A_2B_, and A_3_) [32,35]. P2 receptors fall into two families, P2Y and P2X [36,37]. The P2Y family of G-protein-coupled receptors has eight subtypes (P2Y1, P2Y2, P2Y4, P2Y6, P2Y11, P2Y12, P2Y13, and P2Y14) [38,39]. The P2X family are nonselective cationic ligand-operated channel receptors. Opening of the pore, which is permeable to Na^+^, K^+^, and Ca^2+^, results in depolarization of the cellular membrane [40]. P2X receptors have seven subtypes (P2X1 through 7) and form homo- or hetero-trimeric complexes [40,41]. Various purinergic receptors are involved in the physical, physiologic, neurologic, and immunologic homeostasis of the gastrointestinal tract, and these receptors are expressed in the nervous, epithelial–mesenchymal, and immune systems [42,43,44].

The characteristics of P2X7R differ from those of other P2X receptors. P2X7R contains a cysteine-rich extracellular domain, a short intracellular N-terminus, and a long intracellular C-terminal domain [45,46]. The C-terminal domain of P2X7 contains a conserved lipopolysaccharide-binding motif, and directly coordinates signaling related to macrophage function and lipopolysaccharide action [46]. In addition, proteomic assays revealed that the P2X7R C-terminal region interacts with several proteins, including heat-shock proteins; these interactions may be important for efficient receptor activity [47,48]. Brief exposure of P2X7R to eATP or a nonselective agonist causes cellular depolarization and a massive influx of Ca^2+^, thus initiating inflammatory signal transduction through pore formation by mitogen-activated protein kinase and activation of nuclear factor κB (NF-κB) [49,50]. Therefore, among the P2 receptors, P2X7R is involved mainly in the induction of inflammatory responses.

In addition, eATP acts as an inter-species messenger between commensal bacteria and various host cells, and is essential to maintaining the intestinal ecosystem. It is gradually becoming clearer that eATP also acts as a disease-induction and inflammation-exacerbating factor.

## 2. eATP as an Inflammatory Mediator in Intestinal Inflammation

Extracellular nucleotides (e.g., ATP and UTP), which trigger inflammation, are released from apoptotic cells through hemichannels (e.g., Panx1 and Conx43) [51,52,53]. In the initiation of inflammation, extracellular purines (adenosine, ADP, and ATP) and pyrimidines (UDP and UTP), released from various sources as described earlier, stimulate purinergic receptors in both an autocrine and a paracrine manner. For example, mice with hapten-induced colitis had increased levels of eATP in the intestinal lumen [18]. Moreover, eATP concentrations also increase during other intestinal inflammatory states, including graft-versus-host disease and irradiation-induced abdominal fibrosis [54,55].

These extracellular purines and pyrimidines are involved in the pathogenesis of IBD. One UDP receptor, P2Y6R, is highly expressed on the T-cells that infiltrate the inflamed colonic tissues in active IBD, but not on the T cells in unaffected intestine, thus suggesting that P2Y6R plays a role in the pathogenesis of IBD [56]. Expression of both P2Y6R and the ATP/UTP receptor P2Y2R increased in the colonic mucosa during colitis [57]. P2Y2R and P2Y6R are involved in the expression of intercellular adhesion molecule 1 (ICAM-1) and chemokine C-X-C motif ligand 8 (CXCL8), respectively, implying that these receptors promote the accumulation of inflammatory cells [57]. It was recently reported that eATP stimulates P2X4R among several P2X receptors, and promotes secretion of CXCL5 from macrophages, which binds to CXCR2 selectively expressed in neutrophils [58]. It is still a controversial issue that P2X4R interacts with P2X7R [59]. However, both P2XRs are considered to be potential drug targets for various inflammatory disorders [60]. Since P2X7R was cloned from rat brain in 1996 [61], its multifunctional roles were reported in a variety of cells, including neural cells, mast cells, macrophages, fibroblasts, epithelial cells, lymphocytes, erythrocytes, and erythroleukemia cells [4,44,62,63]. The eATP-P2X7R pathways play important roles in the immune responses of inflammatory regulation, such as chemotaxis and activation of immune cells. In particular, P2X7R stimulation leads to the release of pro-inflammatory mediators, including IL-1β and IL-6 [64].

Activation of the nucleotide-binding oligomerization domain (Nod)-like receptor family protein 3 (NLRP3) inflammasome activates caspase-1, which is required for the secretion of IL-1β [25,65,66]. In human macrophages, IL-1β production depends on the activation of both the Toll-like receptor (TLR) and P2X7R/NLRP3 pathways [67]. In contrast, dendritic cells (DCs) derived from either murine spleen or bone marrow can secrete substantial amounts of mature IL-1β after stimulation by the TLR pathway alone, in the absence of an ATP signal [68]. These results imply that the underlying mechanism of P2X7R signaling differs among cell types.

Blocking the P2X7R pathway using a specific antibody (1F11 monoclonal antibody) suppressed mast-cell activation and degranulation in colonic tissues, and consequently prevented the development of intestinal inflammation [18]. In addition, deficiency of CD300f, which inhibits mast cells, augmented eATP/P2X7R mast-cell-dependent inflammation [69]. Furthermore, eATP/P2X7R-mediated activation of mast cells not only induces degranulation and inflammatory cytokines, but also chemokines and leukotriene B4 (LTB4) to recruit inflammatory cells (e.g., neutrophils) and subsequently exacerbate intestinal inflammation [18] (Figure 1). Therefore, targeting eATP-mediated mast-cell activation might be a promising novel strategy for the prevention and treatment of intestinal inflammation [70]. In another setting, eATP-P2X7R induced the death of regulatory T cells (Treg), which suppress colitis [63,71]. Moreover, eATP released from commensal bacteria activates a unique subset of lamina proprial antigen-presenting cells (CD70^high^CD11c^low^ cells), leading to the differentiation of Th17 cells (Figure 1). Collectively, these findings show that eATP disrupts the regulatory–inflammatory T-cell balance in the intestinal compartment and initiates intestinal inflammation. Data from both mouse and human studies suggest eATP/P2X7R pathways as promising therapeutic targets for overcoming IBD, and clinical trials for validation of therapeutic targets should be conducted in the future [72,73,74].

Several animals experiments revealed that eATP has not only short-term effects, but also long-term trophic roles; it affects cell proliferation, differentiation, motility, and even death in the chronic inflammatory phase [1]. Blockade of the P2X7R/NLRP3 inflammasome signaling pathway using a P2X7R antagonist suppressed chronic inflammation and fibrotic processes in the pancreas [75]. Recently, P2Y2R and P2X7R were shown to be involved in pulmonary fibrosis [76]. Moreover, an inhibitor of Panx1-mediated eATP release prevents liver and skin fibrosis [77]. These observations show that P2X7R is involved in chronic inflammation, such as fibrosis in various tissues (e.g., lung, kidney, and pancreas) [78]. In this regard, radiation-induced injury of small-intestinal epithelial cells led to increased eATP release due to cryptal cell death [55]; increased eATP release in the cryptal region occurred for at least several weeks. In addition, eATP stimulated excess collagen expression by myofibroblasts positive for α-smooth muscle actin and led to the induction of eosinophils via the release of granulocyte-macrophage colony-stimulating factor. This chronic fibrogenic loop led to irradiation-induced intestinal fibrosis [55]. Although fibrosis typically is a component of healing of injured tissue, accumulated evidence suggests that eATP is involved in detrimental fibrosis rather than healing in the gut.

## 3. Resolution of Inflammation

Once released—whether through secretion, degranulation, hemichannel-dependent release, the OMV pathway, or cell death—eATP is rapidly hydrolyzed to ADP, AMP, and adenosine by cell-surface enzymes such as the ecto-nucleoside triphosphate diphosphohydrolase 1 (ENTPDase1, CD39) and ecto-5′-nucleotidase (CD73) [2,79]. Enhanced expression of CD39 and CD73 and the production of extracellular adenosine terminate inflammatory responses. CD39 and CD73 are expressed not only in a variety of tissues, but also in various immune cells, including monocytes, neutrophils, DCs, and B- and T-cell subsets [80]. The expression levels of CD39 and CD73 differ depending on the intestinal environment and condition such as cytokine level, and thus, differently influence the immune response.

Low-level CD39 expression and consequently decreased adenosine production might hamper immunosuppression of inflammation in IBD. For example, intestinal inflammation in murine colitis models was increased in mice defective in CD73 and CD39 compared with wild-type mice [81,82]. Patients with Crohn’s disease (CD) often carry single-nucleotide polymorphisms associated with decreased CD39 expression [81]. CD39 primarily is expressed by activated Tregs and exerts an anti-inflammatory function by reducing pro-inflammatory eATP [83,84,85] (Figure 1). Furthermore, deletion of CD39 in Tregs reduces their immune-suppressive properties [86]. Among Treg (which are CD4^+^ CD25^high^) subsets are cells that express high levels of CD39 [87]; this CD4^+^ CD25^high^ CD39^+^ population suppresses pathogenic Th17 cells [88]. In addition, mesenchymal stromal cells promote CD39 expression on activated T cells and increase adenosine production to suppress excess immune activation in both an autocrine and paracrine manner [89].

Several reports also describe the function of CD39 in mast cells [90,91]. CD39 on the mast-cell surface suppresses the eATP reaching high levels and negatively regulates eATP/P2X7R-mediated cell death and the release of IL-1β [90]. Also, cardiac mast cells showed that CD39 on the cell surface suppresses eATP/P2X7R-mediated renin release which controls the activation of the renin–angiotensin system, thus ultimately exerting a cardioprotective effect [91]. Furthermore, ecto-nucleotide pyrophosphatase–phosphodiesterase 3 (E-NPP3), also known as CD203c, is involved in the clearance of eATP [92]. CD203c is highly expressed on activated basophils and mast cells, and is a useful biomarker for the diagnosis of allergic diseases [93]. The deficiency of CD203c led to elevated levels of serum eATP and pathology consistent with mast-cell-dependent allergic inflammation [92].

One of the eight ENTPDases, ENTPDase7, is selectively and highly expressed on small-intestinal epithelial cells [94]. Furthermore, eATP concentrations in the lumen and the number of IL-17-producing Th17 cells in the lamina propria were increased in the small intestines of ENTPDase7-deficient mice. In addition, ENTPDase7-deficient mice showed increased Th17 cell numbers and high tolerance to the intestinal pathogen, *Citrobacter rodentium* [94].

CD73 is a membrane-bound glycoprotein that hydrolyzes extracellular nucleoside monophosphates into bioactive nucleoside intermediates [95]. In humans, CD73 is expressed on subsets of T and B cells, on germinal center follicular DCs, and on thymic medullary reticular fibroblasts and epithelial cells [96]. In CD73-deficient mice, the production of extracellular adenosine is reduced in many tissues (e.g., colon, lung, liver, muscle, heart, and kidney), and interferon gamma (IFNγ)-producing activated T cells are significantly increased [97]. Both Tregs and stromal cells express CD73, and the extracellular adenosine produced in response to CD73 activation is related to the immunosuppressive roles of these cells [98,99]. A co-culture of CD39-expressing stromal cells and CD73-expressing T lymphocytes increased adenosine levels, and the CD39-expressing stromal cells inhibited the proliferation of activated T cells [89]. Pericellular accumulation of adenosine leads to immunosuppression through adenosine A_2A_ receptors [89,100]. Murine Treg cells co-express CD39 and CD73, and promote the production of extracellular adenosine and immunosuppression [84,98]. Interestingly, Treg expression levels of CD39 and CD73 differed among patients with psoriasis vulgaris, pustular psoriasis, and erythrodermic psoriasis, suggesting this difference in expression pattern as a pathologic factor [101]. Similarly, patients with different forms of IBD (ulcerative colitis (UC) and CD) show unique profiles of purinergic receptor genes or other genes involved in purine metabolism such as CD73 expression [102]. Both colonic mucosal biopsy tissue and peripheral blood mononuclear cells from patients with CD showed abnormal (upregulated) expression of A_2A_R, but in peripheral blood mononuclear cells from patients with UC, the expressions of both A_2A_R and A_2B_R were downregulated [102]. These differences in purinergic receptor genes or purine metabolism related genes dysregulation between CD and UC may contribute to the distinctive pathology of these two forms of IBD.

Extracellular adenosine regulates intracellular cyclic AMP (cAMP) levels though adenosine receptors [103]. Among the four adenosine receptors, A_1_R and A_3_R signaling reduces intracellular cAMP levels, whereas A_2A_R and A_2B_R signaling increases cAMP levels through the activation of adenylyl cyclase, and modulates immunosuppression [104,105]. A2_A_R and A2_B_R are upregulated in response to the activation of immune cells—that is, adenosinergic signaling mediates the suppressive effects of T cells, DCs, neutrophils, macrophages, and other immune cells [103]. In this regard, the expressions of CD39 and CD73 differ between M1 and M2 macrophages. In particular, M1 macrophages tend to accumulate ATP, whereas M2 macrophages typically rapidly convert ATP to adenosine [106]. The hydrolysis of eATP to adenosine that is mediated by CD39 and CD73 on the macrophage surface induces the downregulation of inflammatory cytokines and the production of anti-inflammatory cytokines and growth factors through P1 signaling [107].

The expressions of A_2A_R and A_2B_R are induced not only by inflammatory hypoxia; A_2A_R is also induced by tumor necrosis factor alpha (TNFα), IL-1β, and NF-κB [108], and A_2B_R is highly transcriptionally upregulated by hypoxia-inducible factor-1α (HIF-1α) and IFNγ [109]. A_2A_R has high affinity for adenosine, whereas A_2B_R is a low-affinity receptor [110,111]. The adenosine-receptor-mediated pathways have both immunosuppressive and pro-inflammatory effects. For example, intracellular cAMP elevated through A_2A_R on mast cells suppresses histamine release due to degranulation, whereas the adenosine-induced deamination of inosine promotes the degranulation of mast cells through A_3_R [112]. In contrast, other studies showed that the inosine/A_2_R and inosine/A_3_R pathways can have tissue-protective effects [113].

Oral administration of *Lactobacillus reuteri* to Treg-deficient mice prolonged survival time and reduced systemic inflammation [114]. In particular, oral intake of *L. reuteri* improved villus height and crypt depth, and increased the expression of intestinal nucleoside transporters, including equilibrative nucleoside transporter 1 (ENT1) and concentrative nucleoside transporter 2 (CNT2).

Furthermore, *L. reuteri* improved the intestinal microflora, thus increasing the concentration of inosine from microbial origin, and leading to the immunosuppression of intestinal inflammation through A_2A_R [114].

In colitis, TNFα stimulation increases the expression of A_2B_R in epithelial cells [115], which helps protect against loss of the intestinal epithelial barrier [116]. Furthermore, A_2B_R signaling in the DCs of the small intestine indirectly induces Th17 cells [117]; the Th17-associated cytokine, IL-22, exerts tissue-protective effects during colitis [118]. These different roles of A_2B_R in inflammatory responses may result from differences in tissue-specific receptor-signaling mechanisms or from inflammatory-phase-dependent effects.

In summary, purinergic metabolites produced by the intestinal microflora are involved, through multiple purinergic signaling mechanisms, in the promotion or suppression of immune cell function. Crosstalk between the component bacteria of the microflora and various host cells plays an important role in achieving intestinal homeostasis.

## 4. Conclusions

This review focused on the pathophysiologic roles of eATP. The regulation of the initiation and resolution of inflammation through extracellular purinergic pathways is complex. In addition, recent findings suggest that commensal bacteria are the major source of eATP in the intestinal lumen. Furthermore, patients with CD have increased numbers of mucosa-associated adhesive *E. coli* [72]; direct purinergic communication between those bacteria and epithelial cells may lead to eATP-mediated mucosal inflammation. In the future, molecular imaging might be used to precisely elucidate the trafficking of ATP that is released into the luminal and mucosal inflammatory compartments in patients with IBD. Accumulating evidence suggests purinergic signaling as a promising drug target for treating intestinal inflammation, and this possibility warrants further investigation.

## Abbreviations

LP	lamina propria
OMV	outer membrane vesicle
R	receptor
ROS	reactive oxygen species
Tfh cells	follicular helper T cells
